# Carriage prevalence and serotype distribution of *Streptococcus pneumoniae* prior to 10-valent pneumococcal vaccine introduction: A population-based cross-sectional study in South Western Uganda, 2014

**DOI:** 10.1016/j.vaccine.2017.07.081

**Published:** 2017-08-04

**Authors:** Fabienne Nackers, Sandra Cohuet, Olivier le Polain de Waroux, Céline Langendorf, Dan Nyehangane, Donny Ndazima, Deborah Nanjebe, Angela Karani, Elioda Tumwesigye, Juliet Mwanga-Amumpaire, J. Anthony G. Scott, Rebecca F. Grais

**Affiliations:** a Epicentre, 8 rue Saint Sabin, 75011 Paris, France; b Department of Infectious Disease Epidemiology, London School of Hygiene and Tropical Medicine, Keppel Street, WC1E 7HT London, United Kingdom; c Epicentre, Mbarara Research Centre, P.O. Box 1956, Mbarara, Uganda; d Kenya Medical Research Institute-Wellcome Trust Research Programme, Kilifi, Kenya; e Kabwohe Medical Research Centre, P.O. Box 347, Bushenyi, Kabwohe, Uganda; f Mbarara University of Science and Technology, P.O. Box 1404, Mbarara, Uganda

**Keywords:** *Streptococcus pneumoniae*, Nasopharyngeal carriage, Pneumococcal serotypes, Pneumococcal conjugate vaccine, Children, Adult, Uganda

## Abstract

**Background:**

Information on *Streptococcus pneumoniae* nasopharyngeal (NP) carriage before the pneumococcal conjugate vaccine (PCV) introduction is essential to monitor impact. The 10-valent PCV (PCV10) was officially introduced throughout Ugandan national childhood immunization programs in 2013 and rolled-out countrywide during 2014. We aimed to measure the age-specific *Streptococcus pneumoniae* carriage and serotype distribution across all population age groups in the pre-PCV10 era in South Western Uganda.

**Methods:**

We conducted a two-stage cluster, age-stratified, cross-sectional community-based study in Sheema North sub-district between January and March 2014. One NP swab was collected and analyzed for each participant in accordance with World Health Organization guidelines.

**Results:**

NP carriage of any pneumococcal serotype was higher among children <2 years old (77%; n = 387) than among participants aged ≥15 years (8.5%; n = 325) (chi^2^ p < 0.001).

**Results:**

Of the 623 positive cultures, we identified 49 serotypes among 610 (97.9%) isolates; thirteen (2.1%) isolates were non-typeable. Among <2 years old, serotypes 6A, 6B, 14, 15B, 19F and 23F accounted for half of all carriers. Carriage prevalence with PCV10 serotypes was 29.4% among individuals aged <2 years (n = 387), 23.4% in children aged 2–4 years (n = 217), 11.4% in 5–14 years (n = 417), and 0.4% among individuals ≥15 years of age (n = 325). The proportion of carried pneumococci serotypes contained in PCV10 was 38.1% (n = 291), 32.8% (n = 154), 29.4% (n = 156), and 4.4% (n = 22) among carriers aged <2 years, 2–4 years, 5–14 years and ≥15 years, respectively.

**Discussion:**

In Sheema district, the proportion of PCV10 serotypes was low (<40%), across all age groups, especially among individuals aged 15 years or older (<5%). PCV10 introduction is likely to impact transmission among children and to older individuals, but less likely to substantially modify pneumococcal NP ecology among individuals aged 15 years or older.

## 1 Introduction

*Streptococcus pneumoniae* is a leading cause of morbidity and mortality, particularly in young children, especially in low-income settings [1]. The human nasopharynx (NP) is the main reservoir of person-to-person transmission of pneumococci. Nasopharyngeal colonization is very common, usually asymptomatic, but may sometimes lead to the development of pneumococcal disease [2,3].

Pneumococcal conjugate vaccines (PCVs), targeting 10 (PCV10) and 13 (PCV13) pneumococcal serotypes, are steadily being introduced throughout African national childhood immunization programs. Introduction of PCVs is expected to reduce the incidence of severe pneumococcal diseases and to reduce, or eliminate, NP carriage with vaccine-type (VT) pneumococci. By reducing carriage, PCV reduces transmission and therefore confers indirect protection more widely across the entire population [4]. While serotypes not targeted by the vaccine tend to replace eliminated VT pneumococci in the NP following PCV introduction [5], overall net reductions in the burden of invasive pneumococcal disease (IPD) have been observed [6–8]. However, the impact of PCV on IPD tends to vary across countries and settings [8], notably due to differences in the epidemiology of NP carriage and circulating serotypes. Studying nasopharyngeal carriage across all age groups is critical to monitor the indirect impact of PCV among the entire population [4,9] and to predict the impact on disease through transmission dynamic models. Where the collection of highquality IPD incidence data is impracticable, carriage studies provide a valid alternative to assess the population impact of PCV introduction [10,11].

In Uganda, PCV10 was introduced officially in April 2013 and rolled-out countrywide during 2014 [12]. PCV10 includes serotypes 1, 4, 5, 6B, 7F, 9V, 14, 18C, 19F and 23F. Previous NP carriage studies conducted in Uganda were restricted to specific risk groups (HIV positive adults [13]; children with sickle cell disease [14], children attending routine outpatient consultation [15], or children younger than 5 years living in rural villages of East Uganda [16,17]. To our knowledge, there has been no previous evaluation of pneumococcal carriage and serotype distribution across all age groups in Uganda prior to vaccine introduction. Therefore, we conducted a community-based study in Sheema district (South Western Uganda) to measure the age-specific prevalence and serotype distribution of pneumococcal carriage in the pre-PCV10 period and to provide a benchmark against which findings from the post-PCV10 period can be compared.

## 2 Material and methods

### 2.1 Study setting and target population

The study was conducted between January 22 and March 15, 2014 in four rural sub-counties (Kibingo/Sheema town council, Kabwohe-Itendero town council, Kagango and Kigarama) of Sheema North sub-district, located in the south of the Western region of Uganda. The area included 215 rural villages and two small towns (Kabwohe and Itendero) with a total of 76,804 inhabitants [18]. In Uganda there are two dry seasons between December to February and June to August. The Uganda National Expanded Program on Immunization (UNEPI) recommends three primary doses of PCV10, given at 6, 10 and 14 weeks of age (*i.e.* ‘3 + 0’ schedule). However, at the time of the study, PCV10 had not yet been introduced in Sheema district. Although PCV10 was introduced officially in Uganda, PCV13 might also become available subject to out-of-pocket payment in private health services.

### 2.2 Sample size and study design

This was a cross-sectional population-based study using two-stage cluster sampling. The sample size calculations were stratified by age group and based on an expected carriage prevalence of 70% in children aged <5 years, 35% among those aged 5–14 years and 15% among those aged ≥15 years [19–21]. Accounting for a precision of 5%, a design effect of 1.5 and a 10% non-response (refusal or difficulties in processing NP swabs), the target sample size included 538 children aged <2 years, 538 children aged <5 years, 583 children aged 5–14 years, and 327 individuals aged ≥15 years. Considering that children <2 years old represent 40% of the children <5 years old, we aimed to recruit 538 children aged <2 years and 323 (0.6 ∗ 538) children aged 2–4 years, *i.e.* 1771 individuals in total.

We randomly selected 60 clusters from the exhaustive list of villages and town districts, with a probability proportional to their population size. Within each cluster, we randomly selected 30 households from a household census conducted by the village’s health teams and authorities and a specific sample size was targeted in each age group. We only selected one individual within each household to limit intra-household clustering. Where selected individuals were absent, two other attempts were made to contact them (one the same day and, if unsuccessful, the next Saturday).

### 2.3 Data collection

For each participant, study teams collected information on gender, age, household size and composition, history of oral antibiotic treatment and respiratory symptoms (cough, runny nose/sneezing, difficulties breathing, sore throat, otitis) in the two weeks preceding the study.

One NP sample was obtained by experienced nurses from the posterior pharynx of each study participant using a flexible, pediatric-size or adult-size flocked nylon swab (Copan Diagnostics, USA) in accordance with World Health Organization (WHO) guidelines [22].

### 2.4 Laboratory analysis

The NP samples were transported and analyzed according to WHO guidelines [22]. Once collected, NP swabs were inoculated in a skim milk-tryptone-glucose-glycerin (STGG) medium, transported at +4 °C in cool boxes and frozen at the Epicentre laboratory in Mbarara (30 min drive from Sheema district) at −20 °C within eight hours of collection. After vortexing the sample, 50-μL was inoculated onto a selective agar plate of five mg/L gentamicin-Columbia agar with 5% sheep blood and incubated overnight at 37 °C in 5% CO_2_. One colony from the most predominant typical pneumococcal morphology was selected and tested for optochin susceptibility. In case of growth inhibitory zone between seven and 13 mm in diameter, bile solubility was used to confirm pneumococcal identification. Pure pneumococcal isolates were stored at −80 °C in STGG until shipment under negative cold chain to the laboratory of the KEMRI-Wellcome Trust Research Programme, Kilifi, Kenya, for serotyping. There, pneumococcal isolates were first tested by latex agglutination and then serotyped by the Quellung reaction, using polyclonal rabbit antisera (Statens Serum Institut, Copenhagen, Denmark). The strains with inconclusive serotyping by Quellung reaction were tested by polymerase chain reaction (PCR) targeting the genes encoding autolysin (lytA). If lytA-positive, the isolates were then subjected to sequential multiplex PCR to deduce capsular type [23]. We performed internal quality control of STGG, gentamicin blood agar to ensure sterility and the ability to support pneumococcal growth. Serotyping was monitored by a twice-monthly internal quality assurance scheme, using pneumococcal reference strains. Additionally, 10% of all serotyped isolates were randomly selected and tested by multiplex PCR as Quellung reaction quality control.

### 2.5 Data analysis

Data were double-entered using Voozanoo (www.voozanoo.net, Epiconcept, Paris, France). To adjust for clustering, standard errors were computed by using the linearized variance estimator. To account for the unequal intra-household sampling probabilities, analyses were weighted on the household size. Reported proportions are weighted unless otherwise specified. Results combining all age strata were also weighted to account for the age-stratified design. The prevalence of NP carriage with *S. pneumoniae* is presented with their associated 95% confidence intervals (95%CI). Associations between *S. pneumoniae* NP carriage and participant characteristics (sex, history of oral antibiotic treatment and respiratory symptoms) were analyzed by logistic regression, adjusting for age group (<2 years; 2–4 years; 5–14 years; ≥15 years). Data analysis was conducted using STATA version 13 (Stata Corporation, College Station, Texas, USA).

### 2.6 Ethics

Ethical clearance was obtained from the Ethical review board of Médecins Sans Frontières, the Faculty of Medicine Research & Ethics Committee of the Mbarara University of Science and Technology (MUST), the MUST – Research and Ethics Committee, the Uganda National Council for Science and Technology and the London School of Hygiene and Tropical Medicine. All participants (or caregivers for individuals younger than 18 years of age) provided written informed consent before participation. In addition, children (from 8 to 17 years old) provided assent prior to participation.

## 3 Results

### 3.1 Study population

From 1771 randomly selected households, individuals from 67 (3.8%) households refused to participate, 156 (8.8%) could not be contacted after two visits, and 202 (11.4%) did not meet the sampling age requirements. In total, 1346 households participated in the study, including 387 children <2 years old, 217 aged 2–4 years old, 417 aged 5–14 years old and 325 aged ≥15 years old. These represented, respectively, 72%, 67%, 72% and 99% of the targeted samples. The median household size was 5 (range 1–18) with a median of two individuals younger than 15 years old per household (range 0–11). Overall, 698 (56.3%, weighted on the household size and to account for the age-stratified design) participants were female. Respectively, 168 (12.2%, weighted) and 182 (12.8%, weighted) of the participants reported oral antibiotic intake and respiratory symptoms in the two weeks preceding the study (Table 1).

### 3.2 Carriage prevalence and serotype distribution

Of the 1346 NP swab collected, 626 suspected *S. pneumoniae* were isolated on culture, but three were not confirmed as *S. pneumoniae* by PCR lytA, leaving 623 positive samples. NP carriage prevalence was higher among children <2 years old (77.0%) than among participants aged ≥15 years old (8.5%) for any pneumococcal serotype (chi^2^ p < 0.001) (Table 1). Carriage prevalence with PCV10 serotypes was 29.4%, 23.4%, 11.4%, and 0.4% among individuals aged <2 years (n = 387), 2–4 years (n = 217), 5–14 years (n = 417) and ≥15 years (n = 325), respectively (Table 1).

We identified 49 serotypes among 610 (97.9%) isolates, of which 17 could be serotyped only by multiplex PCR. Thirteen (2.1%) isolates were non-typeable (Table 2). Of 63 randomly selected isolates tested by multiplex PCR as Quellung reaction quality control, 10 isolates were of serotypes not available for confirmation under the PCR schema. Of the 53 remaining isolates, we observed 100% concordance between the serotypes identified by Quellung reaction and by PCR.

**Fig. 1:**
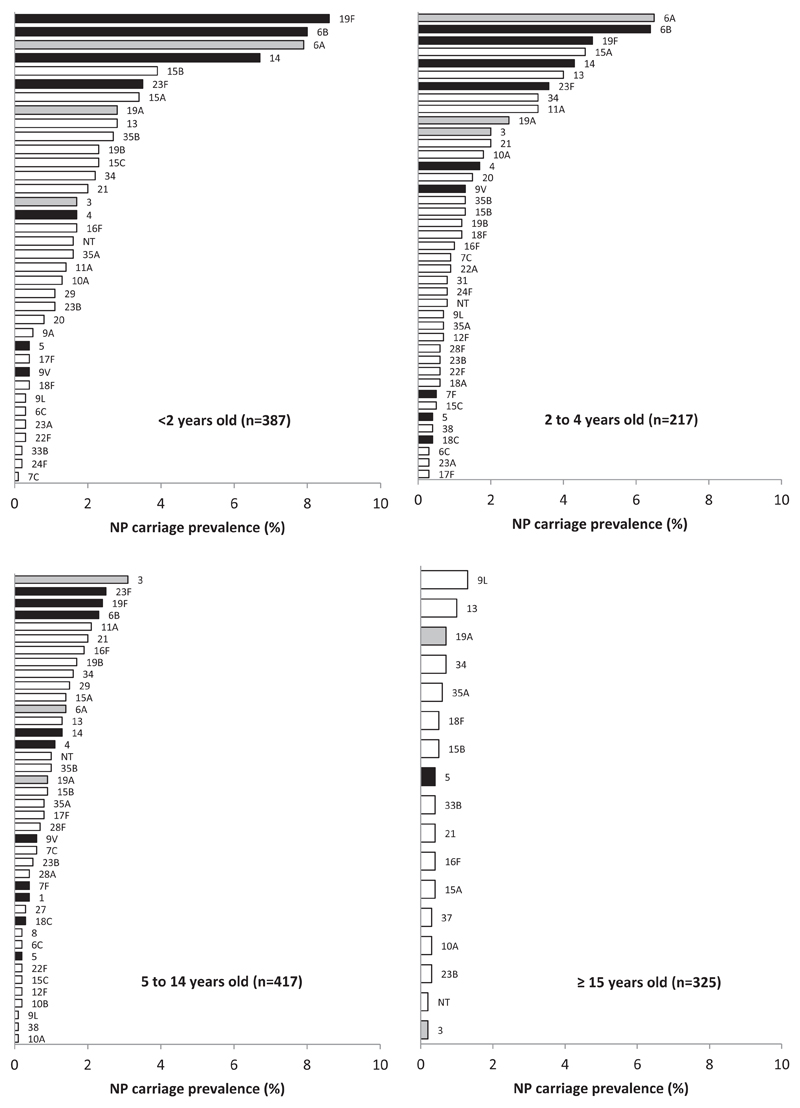
Age-specific serotype NP carriage prevalence. Sheema North Sub District, Uganda, Jan–March 2014. Black = PCV10 serotypes; Grey = PCV13 additional serotypes; White = Non vaccine serotypes; NT = Non-Typeable; NP = Nasopharyngeal; Carriage prevalence are weighted on the household size.

The age-specific serotype distributions are presented in Fig. 1. Among participants <2 years old, six serotypes (6A, 6B, 14, 15B, 19F and 23F) accounted for 50.1% of the carriers. NP carriage prevalence and serotype distributions across six age groups are presented in the supplementary material.

Of all carried pneumococci, PCV10 serotypes (identified by either Quellung reaction or PCR) among carriers <2 years old were 38.1% (n = 291, 95% CI 31.8–44.9), 32.8% (n = 154, 95% CI 25.5–41.0) among carriers 2–4 years old, 29.4% (n = 156, 95% CI 22.2–37.9) among carriers 5–14 years old and 4.4% (n = 22, 95% CI 0.5–29.7) among older carriers (p=0.011). Considering PCV13 serotypes, these proportions were 54.3% (n = 291, 95% CI 47.9–60.5), 48.2% (n = 154, 95% CI 40.4–56.1), 43.4% (n = 156, 95% CI 34.9–52.2) and 15% (n = 22, 95% CI 3.9–43.4) (p = 0.007).

There was no difference in NP carriage of *S. pneumoniae* for female gender (crude OR 0.69, 95% CI 0.54–0.87; weighted and age-adjusted OR 0.83, 95% CI 0.63–1.09), history of oral antibiotic treatment (crude OR 1.16, 95% CI 0.83–1.61; weighted and age-adjusted OR 1.09; 95% CI 0.68–1.76) or respiratory symptoms (crude OR 1.41, 95% CI 1.01–1.96; weighted and age-adjusted OR 1.17; 95% CI 0.77–1.77) in the previous two weeks.

## 4 Discussion

This is the largest community-based study reporting *S. pneumoniae* NP carriage and serotype distribution across all population age groups in the pre-PCV era in Uganda. Studies of a similar size have been conducted in Kenya [19,24,25], but not elsewhere in East Africa.

An estimated 70% of children under 5 years old carried *S. pneumoniae*, which is slightly higher than the 56% pneumococcal carriers reported among 1723 children under 5 years old in a rural community of Eastern Uganda, mostly during the dry season from 2008 to 2011 [16,17]. However, this is consistent with the community-based estimations from large Kenyan carriage studies [20,24]. In a meta-analysis pooling pre-vaccine data from low income countries (including Kenya, Tanzania, The Gambia, and Bangladesh) [26], carriage prevalence in healthy children under 5 years old was estimated at 64.8% (95% CI: 49.8%–76.1%) with substantial heterogeneity from 35% in Tanzania [27] to over 90% in the Gambia [28]. Besides the methodological differences across studies, variations in carriage estimates may result from differences in climates and season [20,25], burden of underlying immunosuppression [20], crowding or social mixing patterns influencing transmission.

We found no evidence of a difference in pneumococcal carriage prevalence according to gender, recent respiratory symptoms or antibiotic intake. However, this study was not designed to capture differences according to environmental or individual factors. By sampling participants over a large rural and semi-urban area, we intended to capture the overall carriage characteristics in the community, thereby providing an important baseline for post-PCV impact assessments.

The most frequent serotypes found in the present study were comparable to those previously reported in carriage studies in several African countries [26] and, with the exception of the serotype 29 that was less frequent in our study, in children of East Uganda (19F, 23F, 6A, 29, and 6B) [16]. In Sheema district, the proportions of serotypes included in PCV10 or PCV13 were low. We found that only 38.1% of all isolates were included in PCV10 among children under 2 years old and 32.8% among children aged 2–4 years, similar to studies in Kenya and Tanzania where 42–56% of serotypes carried by unvaccinated children under 5 years old were included in the PCV10 [20,24,27]. Estimations reported from East Uganda [16] were comparable, with only half of the circulating pneumococcal serotypes among children under 5 years old covered by available PCVs (42% for PCV10 and 54% for PCV13). However, both in East Uganda [16] and in Sheema, the NP carriage of serotypes 1 and 5 was rare, possibly as a result of their short duration or low carriage density [29]. If these serotypes are substantial causes of IPD in Uganda as they are overall in Africa [30], carriage studies might be of limited value to assess the impact of PCV on their related disease burden in this setting.

Children remain the key drivers of *S. pneumoniae* transmission, and consequently, have long been the focus of carriage studies. However, modelling of PCV impact requires information on carriage prevalence (and ideally contacts and transmission probabilities) for all age groups – not just the vaccinated age group. Our study is the first to report carriage and serotype distribution among Ugandan adults from the general population prior to vaccine introduction. As expected, age appears as a major determinant of carriage prevalence. We observed lower pneumococcal carriage prevalence among older individuals, with half of the prevalence among those aged older than 5 years. *S. pneumoniae* carriage prevalence was about 9% among those aged ≥15 years, similar to previous observations among Kenyan adults, *i.e.* around 10% [19,25] and around 20% [24]. The proportion of PCV10 serotypes among carriers was also lower among older individuals, representing less than a third of all isolates in participants aged 5–14 years and, although estimated in a relatively small sample, less than 5% of all isolates among participants aged ≥15 years.

The prevalence and distribution of circulating serotypes not included in PCV10, as well as their level of invasiveness, will be key determinants of the overall impact of the vaccine on disease, given that serotype replacement is likely. Included in PCV13 but not in PCV10, serotype 6A was a major circulating serotype in children under 5 years old in Sheema. This serotype is known as a major cause of childhood IPD in Africa [30] and was the second most frequent serotype identified in a series of 30 children under 5 years old with pneumococcal meningitis in Uganda (after serotype 6B and before serotypes 22A, 23F 14 and 19A) [31]. In this series, 46% of *S. pneumoniae* meningitis were due to serotypes included in PCV10 and 70% in PCV13. Serotypes 19A and 3 are also known causes of IPD [30] and are covered by PCV13 but not PCV10; they were found across all age groups. Other non-vaccine serotypes represented a high proportion of the circulating serotypes in Sheema and they deserve post-PCV monitoring as they are potential candidates for serotype replacement disease in the post-PCV era. For example, serogroup 15 and serotype 35B were relatively frequent in Sheema district and they have emerged among IPD and other pneumococcal diseases in the post-PCV era in several high-income countries [32–35].

Non-vaccine serotypes of low prevalence in the pre-PCV era might also unexpectedly increase in the post-PCV era. Notably, serotype 6C has emerged in several parts of the world after PCV introduction [37] and it became the most common serotype carried by Brazilian children three to four years after the introduction of the PCV10 in the country [36–37]. Although reported of low invasive potential [38], its association with multidrug resistance raises a concern [37]. In Brazil, baseline serotypes were similar to Sheema (6B, 19F, 6A, 14, and 23F [36]) and, after routine PCV10 use, there was a clear impact of PCV10 by reducing the NP carriage with PCV10 serotypes [36–37]. Prevalence of carriage of serotypes 6A and 19A remained unchanged but, in addition to the serotype 6C, there was an increase in serotypes 15A, 15B, 15C, and 11A [36–37]. While knowledge is accumulating on the disease potential of non-vaccine serotypes, carriage studies should ideally be repeated to follow changes in serotype-specific prevalence. How these would actually translate into changes in the serotype-specific pneumococcal disease incidence remains unclear, as little replacement in disease has been reported after complete replacement in carriage [26].

We could not measure the density of carriage at the time of the study. Also, we did not assess multiple or co-carriage nor sensitivity to antimicrobial agents, reported as an increasing concern worldwide, including Uganda [15,17]. Despite these limitations, our results provide important information to monitor changes that will result from PCV10 introduction in Sheema district. They also underline that the proportion of serotypes included in PCV10s was low (<40%), across all age groups, especially among individuals aged ≥15 years (<5%). Introduction of PCV10 is thus likely to impact transmission among children and to older individuals, but less likely to substantially modify pneumococcal NP ecology among individuals aged ≥15 years in this area of Uganda.

## Supplementary Material

Supplementary data associated with this article can be found, in the online version, at http://dx.doi.org/10.1016/j.vaccine.2017.07.081.

Supplementary Data 1

## Figures and Tables

**Table 1:** Participant characteristics and age–specific prevalence of pneumococcal NP carriage, by serotype group, Sheema North Sub District, Uganda, January-March 2014.

	<2 years n = 387			2–4 years n = 217			5–14 years n = 417			≥15 years n = 325		
			
Characteristic	n	%	Weighted %^a^	n	%	Weighted %^a^	n	%	Weighted %^a^	n	%	Weighted %^a^
Female	184	47.6	48.6	104	47.9	48.5	188	45.1	45.3	222	68.3	69.1
Oral antibiotic intake (past 2 weeks)	62	16.0	16.0	26	12.0	10.8	43	10.3	10.7	37	11.4	13.2
Respiratory symptoms (past 2 weeks)	73	18.9	19.9	30	13.8	14.0	48	15.5	11.9	31	9.5	11.9

Carriage	n	%	Weighted % (95%CI)^a^	n	%	Weighted % (95%CI)^a^	n	%	Weighted % (95%CI)^a^	n	%	Weighted % (95%CI)^a^

Any pneumococci	291	75.2	77.0 (72.5–81.0)	154	71.0	71.4 (63.8–78.0)	156	37.4	38.7 (33.5–44.3)	22	6.8	8.5 (5.7–12.4)
PCV10 serotypes^b^	117	30.2	29.4 (24.6–34.7)	50	23.0	23.4 (17.5–30.6)	46	11.0	11.4 (8.2–15.6)	1	0.3	0.4 (0.1–2.7)
PCV13 serotypes^c^	164	42.4	41.8 (36.8–47.0)	74	34.1	34.4 (27.9–41.6)	68	16.3	16.8 (12.9–21.6)	3	0.9	1.3 (0.4–4.3)
Non vaccine serotypes	127	32.8	35.2 (30.0–40.9)	80	36.9	37.0 (30.8–43.6)	88	21.1	21.9 (18.0–26.5)	19	5.9	7.2 (4.6–11.1)

a Weighted on the household size.

b Includes serotypes 1, 4, 5, 6B, 7F, 9V, 14, 18C, 19F and 23F.

c Includes three additional serotypes: 3, 6A and 19A.

**Table 2:** Serotype distribution, Sheema North Sub District, Uganda, January–March 2014 (n = 623).

Serotype (n)	<2 years	2–4 years	5–14 years	≥15 years	Total
6B*	34	14	11	0	59
6A**	32	14	7	0	53
19F*	35	10	8	0	53
14*	26	8	6	0	40
23F*	14	8	9	0	31
13	12	9	5	3	29
15A	11	11	6	1	29
19A**	11	6	3	1	21
3**	4	4	12	1	21
15B	13	3	4	1	21
21	8	4	7	1	20
34	8	6	4	2	20
11A	4	8	7	0	19
35B	9	3	5	0	17
16F	5	3	8	1	17
19B	8	2	6	0	16
Non-typeable	5	2	5	1	13
35A	6	2	3	2	13
4*	5	4	4	0	13
10A	6	4	1	1	12
15C	10	1	1	0	12
29	4	0	6	0	10
23B	4	1	2	1	8
9V*	2	3	2	0	7
7C	1	2	3	0	6
20	3	3	0	0	6
18F	2	2	0	1	5
9L	1	1	1	2	5
17F	1	1	3	0	5
28F	0	1	3	0	4
5*	1	1	1	1	4
12F	0	2	1	0	3
6C	1	1	1	0	3
7F*	0	1	2	0	3
22F	1	1	1	0	3
33B	1	0	0	1	2
1*	0	0	2	0	2
38	0	1	1	0	2
23A	1	1	0	0	2
18C*	0	1	1	0	2
22A	0	2	0	0	2
24F	1	1	0	0	2
Others	1	2	4	1	8

One serotype 16F, one serotype 20, one serotype 34 and fourteen serotypes 14 could be serotyped by PCR only.

* Serotypes included in PCV10 (1, 4, 5, 6B, 7F, 9V, 14, 18C, 19F and 23F).

** Additional serotypes included in PCV13 (3, 6A, and 19A).
