# Supplementary material for: Carriage prevalence and serotype distribution of Streptococcus pneumoniae prior to 10-valent pneumococcal vaccine introduction: A population-based cross-sectional study in South Western Uganda, 2014
Source: Vaccine. Author manuscript; Available in PMC 2019 Jul 9. (PMC6616034; doi:10.1016/j.vaccine.2017.07.081)
Supplement: Supplementary Data 1 [file EMS83622-supplement-Supplementary_Data_1.docx]

**Supplementary Table 1: Age–specific prevalence of any pneumococcal nasopharyngeal carriage and household size distribution, Sheema North Sub District, Uganda, January-March 2014.**

| Age group |  | <1 year | | 1year | | 2-4years | | 5-14years | | 15-29years | | ≥30years | |
| --- | --- | --- | --- | --- | --- | --- | --- | --- | --- | --- | --- | --- | --- |
| Household size | Total n | n (carrier) | N | n (carrier) | N | n (carrier) | N | n (carrier) | N | n (carrier) | N | n (carrier) | N |
| 1 | 24 | 0 | 0 | 0 | 0 | 0 | 0 | 0 | 0 | 0 | 7 | 0 | 17 |
| 2 | 77 | 1 | 2 | 15 | 1 | 3 | 5 | 3 | 16 | 0 | 19 | 0 | 34 |
| 3 | 182 | 22 | 31 | 30 | 25 | 14 | 24 | 10 | 45 | 0 | 19 | 2 | 38 |
| 4 | 277 | 33 | 42 | 20 | 43 | 31 | 39 | 27 | 78 | 3 | 32 | 5 | 43 |
| 5 | 230 | 32 | 48 | 51 | 24 | 27 | 37 | 39 | 78 | 3 | 18 | 4 | 25 |
| 6-7 | 365 | 36 | 46 | 17 | 61 | 53 | 73 | 53 | 133 | 2 | 14 | 1 | 38 |
| 8-9 | 130 | 19 | 24 | 5 | 22 | 14 | 24 | 15 | 43 | 0 | 6 | 1 | 11 |
| ≥10 | 61 | 10 | 12 | 0 | 6 | 12 | 15 | 9 | 24 | 1 | 2 | 0 | 2 |
| All households | 1346 | 153 | 205 | 138 | 182 | 154 | 217 | 156 | 417 | 9 | 117 | 13 | 208 |
| Unweighted prevalence | | 74.6% | | 75.8% | | 71.0% | | 37.4% | | 7.7% | | 6.3% | |
| Weighted prevalence^a^ | | 76.0% ^a^ | | 78.2% ^a^ | | 71.4% ^a^ | | 38.7 ^a^ | | 10.6% ^a^ | | 7.3% ^a^ | |

^a^ Weighted on the household size

**Supplementary Table 2: Age–specific prevalence of pneumococcal nasopharyngeal carriage of PCV10 serotypes* and household size distribution, Sheema North Sub District, Uganda, January-March 2014.**

| Age group |  | <1 year | | 1year | | 2-4years | | 5-14years | | 15-29years | | ≥30years | |
| --- | --- | --- | --- | --- | --- | --- | --- | --- | --- | --- | --- | --- | --- |
| Household size | Total n | n (carrier) | N | n (carrier) | N | n (carrier) | N | n (carrier) | N | n (carrier) | N | n (carrier) | N |
| 1 | 24 | 0 | 0 | 0 | 0 | 0 | 0 | 0 | 0 | 0 | 7 | 0 | 17 |
| 2 | 77 | 1 | 2 | 0 | 1 | 2 | 5 | 2 | 16 | 0 | 19 | 0 | 34 |
| 3 | 182 | 7 | 31 | 10 | 25 | 4 | 24 | 4 | 45 | 0 | 19 | 0 | 38 |
| 4 | 277 | 16 | 42 | 12 | 43 | 8 | 39 | 4 | 78 | 0 | 32 | 0 | 43 |
| 5 | 230 | 14 | 48 | 8 | 24 | 10 | 37 | 14 | 78 | 1 | 18 | 0 | 25 |
| 6-7 | 365 | 10 | 46 | 23 | 61 | 16 | 73 | 13 | 133 | 0 | 14 | 0 | 38 |
| 8-9 | 130 | 5 | 24 | 7 | 22 | 7 | 24 | 6 | 43 | 0 | 6 | 0 | 11 |
| ≥10 | 61 | 4 | 12 | 0 | 6 | 3 | 15 | 3 | 24 | 0 | 2 | 0 | 2 |
| All households | 1346 | 57 | 205 | 60 | 182 | 50 | 217 | 46 | 417 | 1 | 117 | 0 | 208 |
| Unweighted prevalence | | 27.8% | | 33.0% | | 23.0% | | 11.0% | | 0.9% | | 0.0% | |
| Weighted prevalence^a^ | | 27.0% ^a^ | | 32.1% ^a^ | | 23.4% ^a^ | | 11.4% ^a^ | | 1.0% ^a^ | | 0.0% ^a^ | |

^a^ Weighted on the household size *Includes serotypes 1, 4, 5, 6B, 7F, 9V, 14, 18C, 19F and 23F

**Supplementary Table 3: Age–specific prevalence of pneumococcal nasopharyngeal carriage of PCV13 serotypes* and household size distribution, Sheema North Sub District, Uganda, January-March 2014.**

| Age group |  | <1 year | | 1year | | 2-4years | | 5-14years | | 15-29years | | ≥30years | |
| --- | --- | --- | --- | --- | --- | --- | --- | --- | --- | --- | --- | --- | --- |
| Household size | Total n | n (carrier) | N | n (carrier) | N | n (carrier) | N | n (carrier) | N | n (carrier) | N | n (carrier) | N |
| 1 | 24 | 0 | 0 | 0 | 0 | 0 | 0 | 0 | 0 | 0 | 7 | 0 | 17 |
| 2 | 77 | 1 | 2 | 0 | 1 | 2 | 5 | 2 | 16 | 0 | 19 | 0 | 34 |
| 3 | 182 | 13 | 31 | 13 | 25 | 7 | 24 | 4 | 45 | 0 | 19 | 1 | 38 |
| 4 | 277 | 21 | 42 | 16 | 43 | 15 | 39 | 11 | 78 | 0 | 32 | 0 | 43 |
| 5 | 230 | 18 | 48 | 11 | 24 | 13 | 37 | 18 | 78 | 1 | 18 | 0 | 25 |
| 6-7 | 365 | 20 | 46 | 29 | 61 | 24 | 73 | 21 | 133 | 0 | 14 | 0 | 38 |
| 8-9 | 130 | 7 | 24 | 8 | 22 | 7 | 24 | 8 | 43 | 0 | 6 | 1 | 11 |
| ≥10 | 61 | 6 | 12 | 1 | 6 | 6 | 15 | 4 | 24 | 0 | 2 | 0 | 2 |
| All households | 1346 | 86 | 205 | 78 | 182 | 74 | 217 | 68 | 417 | 1 | 117 | 2 | 208 |
| Unweighted prevalence | | 42.0% | | 42.9% | | 34.1% | | 16.3% | | 0.9% | | 1.0% | |
| Weighted prevalence^a^ | | 41.9% ^a^ | | 41.7% ^a^ | | 34.4% ^a^ | | 16.8 ^a^ | | 1.0% ^a^ | | 1.4% ^a^ | |

^a^ Weighted on the household size *Includes serotypes 1, 3, 4, 5, 6A, 6B, 7F, 9V, 14, 18C, 19A, 19F and 23F

**Table 4: Serotypes distribution, Sheema North Sub District, Uganda, January- March 2014 (n=623)**

| **Serotype (n)** | **< 1 years** | **1 year** | **2-4 years** | **5-14 years** | **15-29 years** | **≥ 30 years** | **Total** |
| --- | --- | --- | --- | --- | --- | --- | --- |
| 6B* | 16 | 18 | 14 | 11 | 0 | 0 | 59 |
| 6A** | 18 | 14 | 14 | 7 | 0 | 0 | 53 |
| 19F* | 15 | 20 | 10 | 8 | 0 | 0 | 53 |
| 14* | 13 | 13 | 8 | 6 | 0 | 0 | 40 |
| 23F* | 7 | 7 | 8 | 9 | 0 | 0 | 31 |
| 13 | 9 | 3 | 9 | 5 | 2 | 1 | 29 |
| 15A | 6 | 5 | 11 | 6 | 0 | 1 | 29 |
| 19A** | 7 | 4 | 6 | 3 | 0 | 1 | 21 |
| 3** | 4 | 0 | 4 | 12 | 0 | 1 | 21 |
| 15B | 9 | 4 | 3 | 4 | 1 | 0 | 21 |
| 21 | 3 | 5 | 4 | 7 | 1 | 0 | 20 |
| 34 | 2 | 6 | 6 | 4 | 1 | 1 | 20 |
| 11A | 2 | 2 | 8 | 7 | 0 | 0 | 19 |
| 35B | 6 | 3 | 3 | 5 | 0 | 0 | 17 |
| 16F | 5 | 0 | 3 | 8 | 0 | 1 | 17 |
| 19B | 4 | 4 | 2 | 6 | 0 | 0 | 16 |
| Non-Typeable | 0 | 5 | 2 | 5 | 0 | 1 | 13 |
| 35A | 3 | 3 | 2 | 3 | 0 | 2 | 13 |
| 4* | 5 | 0 | 4 | 4 | 0 | 0 | 13 |
| 10A | 2 | 4 | 4 | 1 | 0 | 1 | 12 |
| 15C | 4 | 6 | 1 | 1 | 0 | 0 | 12 |
| 29 | 2 | 2 | 0 | 6 | 0 | 0 | 10 |
| 23B | 2 | 2 | 1 | 2 | 1 | 0 | 8 |
| 9V* | 1 | 1 | 3 | 2 | 0 | 0 | 7 |
| 7C | 1 | 0 | 2 | 3 | 0 | 0 | 6 |
| 20 | 1 | 2 | 3 | 0 | 0 | 0 | 6 |
| 18F | 1 | 1 | 2 | 0 | 0 | 1 | 5 |
| 9L | 0 | 1 | 1 | 1 | 2 | 0 | 5 |
| 17F | 1 | 0 | 1 | 3 | 0 | 0 | 5 |
| 28F | 0 | 0 | 1 | 3 | 0 | 0 | 4 |
| 5* | 0 | 1 | 1 | 1 | 1 | 0 | 4 |
| 12F | 0 | 0 | 2 | 1 | 0 | 0 | 3 |
| 6C | 1 | 0 | 1 | 1 | 0 | 0 | 3 |
| 7F* | 0 | 0 | 1 | 2 | 0 | 0 | 3 |
| 22F | 1 | 0 | 1 | 1 | 0 | 0 | 3 |
| 33B | 1 | 0 | 0 | 0 | 0 | 1 | 2 |
| 1* | 0 | 0 | 0 | 2 | 0 | 0 | 2 |
| 38 | 0 | 0 | 1 | 1 | 0 | 0 | 2 |
| 23A | 0 | 1 | 1 | 0 | 0 | 0 | 2 |
| 18C* | 0 | 0 | 1 | 1 | 0 | 0 | 2 |
| 22A | 0 | 0 | 2 | 0 | 0 | 0 | 2 |
| 24F | 1 | 0 | 1 | 0 | 0 | 0 | 2 |
| Others | 0 | 1 | 2 | 4 | 0 | 1 | 8 |

* Serotypes included in PCV10 (1, 4, 5, 6B, 7F, 9V, 14, 18C, 19F and 23F)

** Additional serotypes included in PCV13 (3, 6A, and 19A)
